# Impact of COVID-19 pandemic on the accuracy of telephone triage of callers with shortness of breath and/or chest discomfort in Dutch out-of-hours primary care: A retrospective observational study

**DOI:** 10.1080/13814788.2024.2430508

**Published:** 2024-11-28

**Authors:** Michelle Spek, Anna S. M. Dobbe, Dorien L. Zwart, Daphne C. A. Erkelens, Geert-Jan Geersing, Esther de Groot, Mathé Delissen, Frans H. Rutten, Roderick P. Venekamp

**Affiliations:** Department of General Practice & Nursing Science, Julius Centre for Health Sciences and Primary Care, University Medical Centre Utrecht, Utrecht University, Utrecht, The Netherlands

**Keywords:** COVID-19, family practice, general practice, primary health care, triage

## Abstract

**Background:**

Anecdotal reports suggest that missed diagnosis in general practice during the first wave of the COVID-19 pandemic contributed to a drop in life-threatening events (LTEs) detected in hospitals.

**Objectives:**

To investigate the impact of the COVID-19 pandemic on the accuracy of urgency allocation by telephone triage of patients with shortness of breath and/or chest discomfort in out-of-hours primary care (OHS-PC). Accuracy is defined as the correct allocation of high urgency to patients with LTEs and low urgency to those without.

**Methods:**

Retrospective observational study with data from callers contacting OHS-PC for shortness of breath and/or chest discomfort, between 1 March and 1 June 2019 (pre-pandemic) and 1 March to 1 June 2020 (first wave COVID-19 pandemic). Sensitivity and specificity of telephone urgency allocation were compared during both periods with LTEs, including acute coronary syndrome, and pulmonary embolism, as the reference.

**Results:**

3,064 adults (1,840 COVID-19 pandemic and 1,224 pre-pandemic, *p* < 0.001) were included in the study. The sensitivity of urgency allocation was similar during and before the COVID-19 pandemic (0.68, 95% CI 0.59 to 0.75 vs. 0.68, 95% CI 0.60 to 0.75, *p* = 0.944). Specificity was slightly higher during the COVID-19 pandemic (0.52, 95% CI 0.50 to 0.55 vs. 0.45, 95% CI 0.42 to 0.48, *p* < 0.001).

**Conclusion:**

Despite a surge in calls from adults with shortness of breath and/or chest discomfort during the COVID-19 pandemic, the accuracy of telephone triage for LTEs in OHS-PC remained similar to the pre-pandemic era. Improvement of telephone triage seems necessary in both periods.

## KEY MESSAGES

Despite a surge in calls during the COVID-19 pandemic, the accuracy of urgency allocation of adults with SOB and/or chest discomfort in OHS-PC for LTE remained similar to the pre-pandemic era.

Both safety and efficacy of telephone triage could be improved at the OHS-PC of patients with SOB and/or chest discomfort.

## Introduction

The COVID-19 pandemic, especially the first waves, placed a substantial burden on health care professionals in both general practice and hospitals, as it led to an increased demand for care and placed a peak load on hospital and intensive care unit capacity, due to the influx of critically ill patients [1–7]. During the first wave of COVID-19, a national lockdown was implemented in the Netherlands, including measures such as social distancing, working from home, and the closure of schools, restaurants, and sports facilities [8]. General practitioners (GPs) had to rapidly adapt their care delivery by postponing non-urgent care and utilising safer, digital alternatives to reduce virus’ transmission. Additionally, many patients avoided seeking medical care or treatment due to fear of contracting COVID-19 or to help relieve the pressure on health care services. This created significant changes in both the organisation of GP care and health care seeking behaviour during this period.

In the Netherlands, GPs serve as the gatekeepers of the healthcare system. This means that in general access to emergency department care typically requires a referral from a GP. However, patients or their representatives may in evident emergency situations call an ambulance directly by 112 (the Dutch emergency number).

In such a pandemic circumstance, adequate telephone triage in general practice is especially important to distinguish those requiring urgent care from non-critically ill patients. However, triage in general practice is challenging. The relatively low prevalence of life-threatening events (LTEs) makes it difficult to achieve optimal accuracy in triage [9, 10]. Accuracy is defined as the ratio of correctly assigning a high urgency to patients with a LTE and a low urgency to those without a LTE, divided by all assessments. This means that patients who need urgent care get it timely, thereby avoiding ‘undertriage’, while those who do not need urgent care get it later, that is, avoiding ‘overtriage’. ‘Undertriage’ may result in irreversible harm or even death, while ‘overtriage’ disarranges the acute care chain by putting undesirable strain on health care resources, incur unnecessary health care costs, create delays for those really needing urgent care, and finally is associated with a potentially preventable climate impact of healthcare utilisation [11, 12].

Telephone triage during the first wave of COVID-19 at OHS-PC was complicated by the overwhelming focus on COVID-19 while there was a lack of diagnostic tests for COVID-19. Moreover, there is a similarity in symptomatology, such as shortness of breath and chest discomfort, between patients with mild to moderate COVID-19 and those suffering from LTE such as acute coronary syndrome (ACS) or pulmonary embolism (PE) [13–15].

Studies in the hospital setting reported a decrease in urgent diagnoses during the first COVID-19 wave [16–18]. Anecdotal reports suggest that the main explanation for this drop in the incidence of LTE in hospitals in that period was due to missed diagnoses in general practice [7, 19]. However, evidence substantiating this assumption is lacking.

To investigate whether the accuracy of telephone triage in OHS-PC during the COVID-19 pandemic was less than before the pandemic, we assessed the accuracy of urgency allocation of telephone triage among adults with shortness of breath and/or chest discomfort in out-of-hours primary care (OHS-PC) comparing the first wave of the COVID-19 pandemic to the same period the year before.

## Methods

### Study design and population

This retrospective observational study was performed with data from OHS-PC in the Utrecht region, the Netherlands, from 1 March to 1 June 2019 (pre-pandemic) and 1 March to 1 June 2020 (the first COVID-19 pandemic wave) [20].

Klik of tik om tekst in te voeren. Outside regular working hours, OHS-PC provides urgent primary care to ensure 24/7 medical access. In the Netherlands, as in many other European countries, OHS-PC is organised in large-scale cooperatives [21]. Under the supervision of a GP, triage nurses assess by telephone the urgency of the patient’s health problem and decide whether the patient should be seen by a GP or by another medical professional, within what time frame, and what type of contact is needed (immediate ambulance, home visit, consultation with a GP or telephone advice) [22].

Since 2011, the Netherlands Triage Standard (NTS) has been implemented in the Dutch OHS-PC setting to assist triage nurses with this triage process [21, 23]. The NTS is a semi-automatic decision support tool consisting of a hierarchically ordered computer algorithm. Triage nurses must choose-based on the suspected most severe symptom – one out of 56 ‘entrance complaints’, including shortness of breath and chest discomfort. After completing on average five questions, the NTS automatically generates an urgency level that is linked to a maximum response time. Urgency levels range from U1 (immediate ambulance deployment), U2 (patient seen as soon as possible, within 1 h), U3 (patient seen within 3 h), U4 (patient seen within 24 h) to U5 (telephone advice) [21, 23–25]. The triage nurses and/or supervising GP may overrule the NTS’s generated urgency level if they consider a lower or higher urgency level more appropriate [24].

During the study periods, we included all adults who contacted the OHS-PC with shortness of breath and/or chest discomfort and from whom follow-up data about the final diagnosis could be retrieved from the patient’s own GP electronic health records (EHR). We excluded those below 18 years of age, and patients living outside the vicinity of Utrecht (since follow-up data could not be retrieved from these patients).

### Data collection

Data were collected from both the OHS-PC setting and the patients’ own GPs. Patient demographics and urgency allocation were collected from the OHS-PC EHR. Specifically, data included age in whole years, gender categorised as male or female, and urgency allocation classified into U1 through U5. This urgency allocation was subsequently stratified into high (U1 and U2) and low (U3, U4, and U5) levels for data analyses. These data were linked to the patients’ own GP EHR for follow-up data about the final diagnosis within 30 days of the index contact with the OHS-PC. Diagnoses were recorded and classified into pre-specified categories (e.g. upper respiratory tract infection, acute coronary syndrome, etc.) whenever possible. During the review of patient records, some unanticipated diagnoses, such as gastrointestinal bleeding, intestinal ischaemia, and hypertensive crisis, were encountered. These cases were discussed with an expert panel of experienced GPs. The expert panel was blinded to the urgency assignments of these patients. The panel determined whether these cases should be classified into a new diagnostic category or would fit within one of the pre-defined categories. In cases where a new category was considered, the panel assessed whether these conditions were potentially life-threatening. LTEs justifying high urgency levels (U1-U2) included the following diagnoses: ACS, acute heart failure, pulmonary embolism, anaphylaxis, thoracic aorta dissection, acute upper airway obstruction, sepsis, gastro intestinal bleeding, intestinal ischaemia, hypertensive crisis, pneumothorax, subcutaneous emphysema, vertebral fracture after trauma and laryngeal fracture. The diagnoses COVID-19, asthma/COPD exacerbation, and pneumonia were classified as either mild or moderate (in which U3-U5 was judged accurate) or severe (justifying U1-U2 and thus classified as LTE). Severe was defined as requiring hospital admission or supplemental oxygen administration at home within 24 h of the OHS-PC index contact.

### Main outcome measures

The primary outcome was the accuracy of telephone urgency allocation of patients who contacted the OHS-PC for shortness of breath and/or chest discomfort during and before the COVID-19 pandemic with LTE as the reference [24].

Secondary outcomes included the number of OHS-PC contacts for shortness of breath and/or chest discomfort, the overall urgency allocation, and the incidence of the final diagnoses in both periods.

### Statistical analysis

Patient demographics before and during the COVID-19 pandemic periods were described descriptively and compared using Pearson’s chi-square test (gender) and the independent sample T-test (age).

Accuracy of telephone urgency (high versus low) allocation during both study periods was expressed as sensitivity, specificity, positive predictive value, and negative predictive value with corresponding 95% confidence intervals (CI) with LTE (yes versus no) as the reference. Pearson’s chi-square test was used to compare sensitivity, specificity, positive predictive value, and negative predictive value between both study periods.

The number of OHS-PC contacts and final diagnoses during both study periods were compared with the Binomial test for both the total sample and stratified for shortness of breath and chest discomfort, respectively.

Relative risks (RRs) with corresponding 95% CIs were calculated to analyse the relation between the study period and (i) urgency allocation, and (ii) LTEs in both the overall sample and stratified for shortness of breath and chest discomfort. Interaction terms were used to assess whether callers with shortness of breath differed from those with chest discomfort.

A p-value of <0.05 was considered statistically significant. All data analyses were performed with SPSS statistics 26.0.

## Results

**Figure 1.**
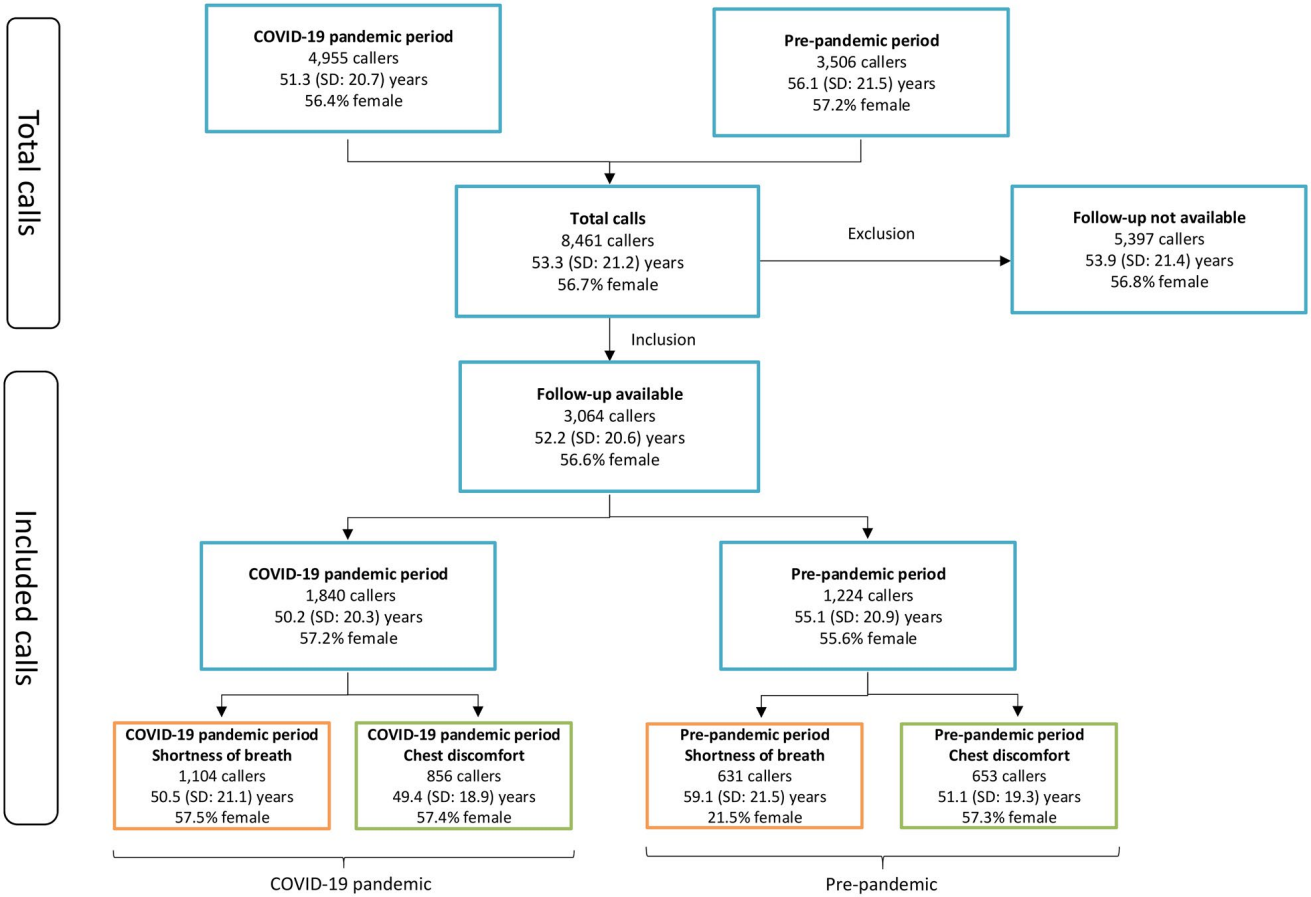
Flowchart of study population. Blue: callers with shortness of breath and/or chest discomfort. Orange: callers with shortness of breath. Green: callers with chest discomfort.

In total, 8,461 patients called OHS-PC for chest discomfort and/or shortness of breath during both study periods. The mean age of these callers was 53.3 (SD: 21.2) years and 56.7% were female. The number of contacts was significantly higher during the COVID-19 pandemic period (4,955 vs. 3,506, *p* < 0.001), driven by the increase of contacts for shortness of breath (2,978 vs. 1,804, *p* < 0.001). Follow-up data were collected from 3,064 callers (36.2%); the mean age 52.2 (SD: 20.6) years and 56.6% were female (Figure 1).

Callers during the COVID-19 pandemic period were on average younger (50.2 (SD: 20.3) vs. 55.1 (SD: 20.9) years, *p* < 0.001) but had a similar gender distribution compared to pre-pandemic callers (female: 57.2% vs. 55.6%, *p* = 0.401).

### Urgency allocation before and during the COVID-19 pandemic

In general, patients who contacted the OHS-PC for shortness of breath and/or chest discomfort during the COVID-19 pandemic less often received a high urgency than those of the pre-pandemic period: 49.3% vs. 56.8%, RR 0.87; 95% CI 0.81 to 0.93. This difference was driven by patients with shortness of breath (RR 0.80; 95% CI 0.72 to 0.90), not by those with chest discomfort (RR 1.01; 95% CI 0.94 to 1.08; *p*-value for interaction <0.001; Supplememntary Material, Table S1).

### Final diagnoses before and during the COVID-19 pandemic

The final diagnoses among patients contacting the OHS-PC for shortness of breath and/or chest discomfort in both study periods are summarised in Table 1 and Supplementary material, Tables S2 and S3.

**Table 1.** Diagnoses of 3,064 callers with shortness of breath and/or chest discomfort, stratified by pre-pandemic period vs. COVID-19 period.

	Pre-pandemic period (2019) *n* = 1,224 (39.9%)	COVID period (2020) *n* = 1,840 (60.1%)	*p*-value
**Life-threatening events**			
**Cardiovascular disorders**			
Acute coronary syndrome	32 (2.6%)	34 (1.8%)	0.152
Acute heart failure	35 (2.9%)	18 (1.0%)	**<0.001**
**Respiratory tract disorders**			
Severe asthma exacerbation	10 (0.8%)	7 (0.4%)	0.137
Severe COPD exacerbation	24 (2.0%)	12 (0.7%)	**<0.001**
Severe COVID-19 infection	N/A	37 (2.0%)	N/A
Severe pneumonia	30 (2.5%)	13 (0.7%)	**<0.001**
**Other disorders**			
Anaphylaxis	10 (0.8%)	3 (0.2%)	**0.009**
Pulmonary embolism	7 (0.6%)	6 (0.3%)	0.396
Other life-threatening events (LTEs)^a^	14 (1.1%)	24 (1.3%)	0.694
**Non-urgent disorders**			
**Cardiovascular disorders**			
Atrial fibrillation or atrial flutter	23 (1.9%)	18 (1.0%)	**0.034**
Stable angina pectoris	15 (1.2%)	10 (0.5%)	**0.040**
Stable heart failure	32 (2.6%)	19 (1.0%)	**<0.001**
**Respiratory tract disorders**			
Bronchitis/bronchial hyperreactivity	12 (1.0%)	15 (0.8%)	0.632
Mild or moderate asthma exacerbation	59 (4.8%)	60 (3.3%)	**0.029**
Mild or moderate COPD exacerbation	42 (3.4%)	48 (2.6%)	0.187
Mild or moderate COVID-19 infection^b^	N/A	279 (15.2%)	N/A
Mild or moderate pneumonia	76 (6.2%)	54 (2.9%)	**<0.001**
Upper respiratory tract infection	100 (8.2%)	204 (11.1%)	**0.008**
**Other disorders**			
Costal contusion or fracture	14 (1.1%)	17 (0.9%)	0.551
Gastro-oesophageal reflux	20 (1.6%)	37 (2.0%)	0.450
Hyperventilation/anxiety/stress	96 (7.8%)	145 (7.9%)	0.970
Shortness of breath due to (existing) cancer	12 (1.0%)	15 (0.8%)	0.632
Unspecified chest pain^c^	267 (21.8%)	310 (16.8%)	**<0.001**
Unspecified shortness of breath^d^	128 (10.5%)	251 (13.6%)	**0.009**
Other non-urgent disorders^e^	166 (13.6%)	204 (11.1%)	**0.039**

ACS: Acute coronary syndrome; GP: General practitioner; LTE: Life-threatening disease; N/A: Not applicable; OHS-PC: Out-of-hours services for primary care.

^a^ Thoracic aorta dissection, acute upper airway obstruction, sepsis, gastrointestinal bleeding, intestinal ischaemia, hypertensive crisis, pneumothorax, subcutaneous emphysema, vertebral fracture after trauma, and laryngeal fracture.

^b^ As regular testing on COVID-19 did not start during the first COVID-19 peak, we included suspected and proven COVID-19 infections.

^c^ Cardiac pathology unlikely after cardiologist’s or GP’s diagnostic work-up, including those with musculoskeletal chest pain.

^d^ Cardiac or pulmonary pathology unlikely after cardiologists’, pulmonologists’, or GPs’ diagnostic work-up.

^e^ Amongst others: hay fever, hypertension, palpitations, peripheral vestibular syndromes.

**Table 2.** Accuracy of urgency allocation for detecting LTE of 3,064 callers who called the OHS-PC with shortness of breath and/or chest discomfort, stratified by pre-pandemic period vs. COVID-19 period.

	Pre-pandemic period (2019) (95% CI)	COVID period (2020) (95% CI)	*p*-value
**Total calls**			
Sensitivity	0.68 (0.60-0.75)	0.68 (0.59-0.75)	0.944
Specificity	0.45 (0.42-0.48)	0.52 (0.50-0.55)	**<0.001**
Positive predictive value	0.16 (0.13-0.19)	0.11 (0.09-0.14)	0.011
Negative predictive value	0.90 (0.87-0.93)	0.95 (0.93-0.96)	**0.001**
**Shortness of breath**			
Sensitivity	0.64 (0.54-0.73)	0.60 (0.50-0.70)	0.576
Specificity	0.56 (0.52-0.61)	0.64 (0.61-0.67)	**0.003**
Positive predictive value	0.24 (0.20-0.30)	0.15 (0.12-0.19)	**0.002**
Negative predictive value	0.88 (0.84-0.91)	0.94 (0.92-0.95)	**<0.001**
**Chest discomfort**			
Sensitivity	0.77 (0.63-0.87)	0.85 (0.72-0.93)	0.277
Specificity	0.34 (0.30-0.38)	0.34 (0.30-0.37)	0.965
Positive predictive value	0.09 (0.07-0.12)	0.08 (0.06-0.11)	0.506
Negative predictive value	0.94 (0.90-0.97)	0.97 (0.94-0.99)	0.131

LTE: Life-threatening disease; OHS-PC: Out-of-hours services for primary care.

Although the occurrence of LTEs remained stable during both periods in terms of absolute numbers (154 vs 162), LTEs occurred relatively less frequently during the COVID-19 pandemic: 8.4% vs. 13.2% (RR 0.63; 95% CI 0.51 to 0.78). This was significant in patients contacting the OHS-PC for shortness of breath (9.6% vs. 18.1%, RR 0.53; 95% CI 0.42 to 0.68), but not among patients contacting for chest discomfort (6.3% vs. 8.0%, RR 0.79; 95% CI 0.55 to 1.14; p-value for interaction = 0.076; Supplementary Material, Table S4).

### Accuracy of urgency allocation

Callers with LTE received a high urgency allocation in 67.7%, while those without LTE received a high urgency allocation in 50.5%. This was comparable in both study periods (67.5% vs. 47.7% and 67.9% vs. 55.1%, respectively; p-value for interaction = 0.218). In 102 (32.3%) callers with LTE, a low urgency was assigned, most often U3 (75.5%), followed by U5 (15.7%) and U4 (8.8%). In 1,359 (49.5%) callers without a LTE, a low urgency was assigned.

Sensitivity of urgency allocation was comparable during the COVID-19 pandemic and pre-pandemic period: 0.68 (95% CI 0.59–0.75) vs. 0.68 (95% CI 0.60–0.75), *p* = 0.944, while specificity was higher during the COVID-19 pandemic: 0.52 (95% CI 0.50–0.55) vs. 0.45 (95% CI 0.42–0.48), *p* < 0.001 (Table 2). The positive predictive value (0.11 (95% 0.09–0.14) vs. 0.16 (95% CI 0.13–0.19)) and negative predictive value (0.95 (95% CI 0.93–0.96) vs. 0.90 (95% CI 0.87–0.93) were comparable during both study periods (Table 2).

Stratified analyses for gender (females and males) and age categories (<40, 40–59, 60–79, >80 years) yielded comparable results (data not shown).

## Discussion

### Main findings

During the first wave of the COVID-19 pandemic more adults with shortness of breath and/or chest discomfort contacted the OHS-PC than during a similar pre-pandemic period. While the absolute number of LTEs remained stable over time (154 vs. 162), LTEs occurred relatively less frequently among callers during the COVID-19 pandemic than pre-pandemic (8.4% vs. 13.2%, respectively). The surge in calls of patients with shortness of breath in our study was also reported in a previous study using data of office hours general practice care during the first wave of the COVID-19 pandemic, possibly driven by fear of having acquired a SARS-CoV-2 infection [26].

Despite a surge in calls during the COVID-19 pandemic, the safety of telephone triage of callers with shortness of breath and/or chest discomfort in OHS-PC for LTE in terms of sensitivity remained similar to the pre-pandemic era, while specificity as measure of efficiency was slightly higher during the COVID-19 pandemic.

In both study periods, however, about two-thirds of adults who contacted OHS-PC with shortness of breath and/or chest discomfort and who showed to have a LTE received a high urgency, which indicates ‘undertriage’. This was around 50% in the patients without LTE, which indicates ‘overtriage’. These data suggest that there is room for improvement of telephone triage of callers with shortness of breath and/or chest discomfort in terms of both safety and efficiency.

### Strengths and limitations

This is the first study to assess the impact of the COVID-19 pandemic on the accuracy of telephone triage in patients contacting OHS-PC with shortness of breath and/or chest discomfort. We did not have strict exclusion criteria which ensures a representative real-life study population. Data were derived from three OHS-PC centres in the Netherlands, including both urban and rural areas. Our results are likely generalisable to other countries with a similar primary health care system, e.g. Scandinavian countries, Germany, and the United Kingdom [27].

Some limitations deserve further attention. As per routine practice, not all patients in our study sample were transferred to the hospital for further diagnostic work-up. This may have led to some diagnostic misclassifications. To reduce such misclassification as much as possible, we collected data about the final diagnosis from the patient’s primary care electronic health record up to 30 days after the index contact at the OHS-PC in both study periods. By doing so, we included a diagnostic period in which additional testing could have been performed, if necessary, to determine the final diagnosis after the index contact at the OHS-PC. It is therefore unlikely that any misclassification of LTEs has substantially influenced our main findings.

Moreover, we compared only a pre-pandemic period with the first COVID-19 wave. Therefore, it is still unknown whether diagnostic accuracy differed during other periods of the COVID-19 pandemic. However, because the lack of testing was primarily a problem during the first wave, patient anxiety decreased during the pandemic, and health care professionals were more accustomed to this pandemic situation later in the pandemic, the potential impact on triage was likely greatest during this first wave. Since we looked during this first wave period and found no effect, it is unlikely that there would be a difference later in the pandemic.

Another limitation is that we included 36.2% of all patients with shortness of breath who called OHS-PC because in the remaining 65.8% we could not retrieve follow-up information on the diagnosis of the patient’s GP because the GP did not want to cooperate. The mean age was 52.2 (SD: 20.6) years vs 53.9 (SD: 21.4) years; *p* < 0.001, and the percentage of females was 56.6% vs 56.8%; *p* = 0.806. Although the age difference is statistically significant, it is unlikely that a 1.7-year age difference is clinically relevant. Thus, this selection likely did not cause selection bias, more so because the willingness of GPs to cooperate seems not to be related to the eligibility of triage conversations nor with the medical outcome of individual callers.

### Comparison with existing literature

Compared to the pre-pandemic era, our study showed a 41.3% increase in OHS-PC contacts for shortness of breath and/or chest discomfort during the COVID-19 pandemic. This is in line with another Dutch and Belgian study which observed a 40% increase in OHS-PC telephone consultations for shortness of breath and chest discomfort during the COVID-19 pandemic compared to pre-pandemic [28, 29].

Studies in the hospital setting reported a decrease in urgent diagnoses during the first COVID-19 wave, e.g. a 31 to 66% relative decline in hospital admissions for cardiovascular diseases, most notably ACS [16–18]. Different causes for the lower number of patients with LTEs in hospitals have been described in the literature, including ‘missed’ LTEs in primary care, the alternative help-seeking behaviour of patients, and a real reduction in LTEs during the pandemic due to less exposure to particulate emissions and less (second hand) smoking [30–32]. It remains difficult to explain the decrease in hospital admissions for these urgent diagnoses, but we found neither evidence for a reduction in LTE occurrence in absolute numbers nor evidence of an increase in missed LTEs in OHS-PC during the COVID-19 pandemic. However, according to a US study in the ambulance setting during the first wave of the COVID-19 pandemic, there was a 26.5% decrease in ambulance deployments and a 46.6% relative increase in non-transports to the hospital when an ambulance was at the scene [33]. It is possible that LTEs were missed here, causing a decrease in the absolute number of LTEs.

Studies evaluating the accuracy of telephone triage in OHS-PC during the COVID-19 pandemic are scarce. Similar to our observations, a study conducted in British ambulance services also described room for improvement in the quality of telephone triage in patients with suspected COVID-19. Telephone triage in this study had a sensitivity of 0.74 (95% CI: 0.72 to 0.77) and a specificity of 0.62 (95% CI: 0.61 to 0.62) for death or renal, respiratory, or cardiovascular organ support (serious adverse outcomes) at 30 days after the index contact [34]. However, this study did not include data on the pre-pandemic period.

Compared with diagnostic accuracy studies of telephone triage for callers with chest discomfort from our group in OHS-PC during other pre-COVID-19 pandemic periods, sensitivity and specificity are similar (sensitivity of 0.81 (95% CI 0.72–0.88) vs. 0.86 (95% CI 0.81–0.90) and specificity of 0.34 (95% CI 0.31–0.36) vs. 0.34 (95% CI 0.32–0.37)) [35].

### Implications

Future studies should aim at improving both the safety and efficiency of telephone triage of callers with shortness of breath and/or chest discomfort in OHS-PC. This is, however, challenging since typically an increase in safety coincides with a decline in efficiency. This is also illustrated by the differences in sensitivity and specificity for shortness of breath and chest discomfort in our study. While the sensitivity of urgency allocation with LTE as reference among callers with chest discomfort was higher than among callers with shortness of breath (0.81 vs. 0.63, respectively), the specificity was much lower (0.34 vs. 0.61). Therefore, future research is needed to determine which combination of patient demographics, characteristics and symptoms during telephone triage of callers with shortness of breath and/or chest discomfort can accurately predict the presence of LTE. Ultimately, a prediction model for adults contacting OHS-PC with shortness of breath and/or chest discomfort should be developed, validated, and compared to the usual care to further improve the safety and efficiency of telephone triage in these patients.

## Conclusion

Despite a surge in calls from adults with shortness of breath and/or chest discomfort during the COVID-19 pandemic, the accuracy of telephone triage for LTE at the OHS-PC remained similar to the pre-pandemic era. Overall, there seems to be room for improvement in both safety and efficiency of telephone triage in these patients. This improvement devoted to enhancing patient outcomes could potentially be achieved by developing and validating a prediction model for LTEs among callers with shortness of breath and/or chest discomfort. If proven effective, such a model could be incorporated into the existing decision support tools used for telephone triage in OHS-PC.

## Supplementary Material

Supplemental Material
