# Supplementary material for: Impact of COVID-19 pandemic on the accuracy of telephone triage of callers with shortness of breath and/or chest discomfort in Dutch out-of-hours primary care: A retrospective observational study
Source: Eur J Gen Pract. 2024 Nov 28;30(1):2430508. doi: 10.1080/13814788.2024.2430508 (PMC11610253; doi:10.1080/13814788.2024.2430508)
Supplement: Supplemental Material [file IGEN_A_2430508_SM5549.pdf]

Supplementary data

Table S1. Association between calling OHS-PC during pre-pandemic period (March-May 2019) or first COVID-19 peak in the Netherlands (March-May 2020) and urgency allocation of 3,064 callers with shortness of breath and/or chest discomfort, stratified by entrance complaint.

**Total calls**	**Pre-pandemic period (2019) n=1,224 (39.9%)**	**COVID period (2020) n=1,840 (60.1%)**	**RR (95% CI)**
High urgency	695 (56.8%)	908 (49.3%)	**0.87 (0.81-0.93)**
Low urgency	529 (43.2%)	932 (50.7%)	
**Shortness of breath**	**Pre-pandemic period (2019) n=631 (36.4%)**	**COVID period (2020) n=1,104 (63.6%)**	
High urgency	298 (47.2%)	419 (38.0%)	**0.80 (0.72-0.90)**
Low urgency	333 (52.8%)	685 (62.0%)	
**Chest discomfort**	**Pre-pandemic period (2019) n=653 (43.3%)**	**COVID period (2020) n=856 (56.7%)**	
High urgency	438 (67.1%)	578 (67.5%)	1.01 (0.94-1.08)
Low urgency	215 (32.9%)	278 (32.5%)	

OHS-PC: out-of-hours services for primary care.

Because some callers had both chest discomfort and shortness of breath chosen as entrance complaint, the total number of callers is lower than the number of callers with chest discomfort + the number of callers with shortness of breath

Table S2. Diagnoses of 1,735 callers with shortness of breath, stratified by pre-pandemic period vs. COVID-19 period.

	Pre-pandemic period (2019) n=631 (36.4%)	COVID period (2020) n=1,104 (63.6%)	p-value
Life-threatening events			
Cardiovascular disorders			
Acute coronary syndrome	5 (0.8%)	2 (0.2%)	0.053
Acute heart failure	32 (5.1%)	16 (1.4%)	<0.001
Respiratory tract disorders			
Severe asthma exacerbation	5 (0.8%)	7 (0.6%)	0.074
Severe COPD exacerbation	32 (5.1%)	9 (0.8%)	<0.001
Severe COVID-19 infection	5 (0.8%)	37 (3.4%)	N.A.
Severe pneumonia	32 (5.1%)	13 (1.2%)	<0.001
Other disorders			
Anaphylaxis	9 (1.4%)	1 (0.1%)	<0.001
Pulmonary embolism	4 (0.6%)	4 (0.4%)	0.472
Other life-threatening events (LTEs)*	6 (1.0%)	17 (1.5%)	0.385
Non-urgent disorders			
Cardiovascular disorders			
Atrial fibrillation or atrial flutter	3 (0.5%)	5 (0.5%)	0.947
Stable angina pectoris	2 (0.3%)	0 (0.0%)	0.132
Stable heart failure	29 (4.6%)	16 (1.4%)	<0.001
Respiratory tract disorders			
Bronchitis/bronchial hyperreactivity	11 (1.7%)	12 (1.1%)	0.250
Mild or moderate asthma exacerbation	57 (9.0%)	56 (5.1%)	0.001
Mild or moderate COVID-19 infection**	N.A.	230 (20.8%)	N.A.
Mild or moderate COPD exacerbation	42 (6.7%)	45 (4.1%)	0.018
Mild or moderate pneumonia	62 (9.8%)	47 (4.3%)	<0.001
Upper respiratory tract infection	78 (12.4%)	170 (15.4%)	0.082
Other disorders			
Costal contusion or fracture	5 (0.8%)	5 (0.5%)	0.511
Gastro-oesophageal reflux	1 (0.2%)	5 (0.5%)	0.426
Hyperventilation/anxiety/stress	35 (5.5%)	89 (8.1%)	0.050
Shortness of breath due to (existing) cancer	10 (1.6%)	14 (1.3%)	0.587
Unspecified chest pain***	32 (5.1%)	36 (3.3%)	0.062
Unspecified shortness of breath****	66 (10.5%)	170 (15.4%)	0.004
Other non-urgent disorders*****	84 (13.3%)	98 (8.9%)	0.004
ACS: acute coronary syndrome, GP: general practitioner, LTE: life-threatening disease, N.A.: not applicable, OHS-PC: out-of-hours services for primary care			

* Thoracic aorta dissection, acute upper airway obstruction, sepsis, gastro-intestinal bleeding, intestinal ischaemia, hypertensive crisis, pneumothorax, subcutaneous emphysema, vertebral fracture after trauma, and laryngeal fracture.

** As regular testing on COVID-19 did not start during the first COVID-19 peak, we included suspected and proven COVID-19 infections

*** Cardiac pathology unlikely after cardiologist's or GP's diagnostic work-up, including those with musculoskeletal chest pain

**** Cardiac or pulmonary pathology unlikely after cardiologist's, pulmonologists, or GP's diagnostic work-up

***** Amongst others: hay fever, hypertension, palpitations, peripheral vestibular syndromes

Table S3. Diagnoses of 1,509 callers with chest discomfort, stratified by pre-pandemic period vs. COVID-19 period.

	Pre-pandemic period (2019) n=653 (43.3%)	COVID period (2020) n=856 (56.7%)	p-value
Life-threatening events			
Cardiovascular disorders			
Acute coronary syndrome	27 (4.1%)	32 (3.7%)	0.694
Acute heart failure	4 (0.6%)	3 (0.4%)	0.474
Respiratory tract disorders			
Severe asthma exacerbation	2 (0.3%)	0 (0.0%)	0.187
Severe COPD exacerbation	3 (0.5%)	4 (0.5%)	1.000
Severe COVID-19 infection	N.A.	1 (0.1%)	N.A.
Severe pneumonia	4 (0.6%)	0 (0.0%)	0.035
Other disorders			
Anaphylaxis	1 (0.2%)	2 (0.2%)	1.000
Pulmonary embolism	3 (0.5%)	3 (0.4%)	1.000
Other life-threatening events (LTEs)*	8 (1.2%)	9 (1.1%)	0.808
Non-urgent disorders			
Cardiovascular disorders			
Atrial fibrillation or atrial flutter	21 (3.2%)	15 (1.8%)	0.065
Stable angina pectoris	14 (2.1%)	10 (1.2%)	0.133
Stable heart failure	4 (0.6%)	6 (0.7%)	1.000
Respiratory tract disorders			
Bronchitis/bronchial hyperreactivity	1 (0.2%)	4 (0.5%)	0.397
Mild or moderate asthma exacerbation	3 (0.5%)	6 (0.7%)	0.740
Mild or moderate COVID-19 infection**	N.A.	68 (7.9%)	N.A.
Mild or moderate COPD exacerbation	2 (0.3%)	6 (0.7%)	0.478
Mild or moderate pneumonia	18 (2.8%)	11 (1.3%)	0.039
Upper respiratory tract infection	27 (4.1%)	47 (5.5%)	0.227
Other disorders			
Costal contusion or fracture	11 (1.7%)	12 (1.4%)	0.657
Gastro-oesophageal reflux	19 (2.9%)	34 (4.0%)	0.267
Hyperventilation/anxiety/stress	67 (10.3%)	73 (8.5%)	0.250
Shortness of breath due to (existing) cancer	2 (0.3%)	3 (0.4%)	1.000
Unspecified chest pain***	253 (38.7%)	290 (33.9%)	0.051
Unspecified shortness of breath****	68 (10.4%)	99 (11.6%)	0.480
Other non-urgent disorders*****	91 (13.9%)	118 (13.8%)	0.933
ACS: acute coronary syndrome, GP: general practitioner, LTE: life-threatening disease, N.A.: not applicable, OHS-PC: out-of-hours services for primary care			

* Thoracic aorta dissection, acute upper airway obstruction, sepsis, gastro-intestinal bleeding, intestinal ischaemia, hypertensive crisis, pneumothorax, subcutaneous emphysema, vertebral fracture after trauma, and laryngeal fracture.

** As regular testing on COVID-19 did not start during the first COVID-19 peak, we included suspected and proven COVID-19 infections

*** Cardiac pathology unlikely after cardiologist's or GP's diagnostic work-up, including those with musculoskeletal chest pain

**** Cardiac or pulmonary pathology unlikely after cardiologist's, pulmonologists, or GP's diagnostic work-up

***** Amongst others: hay fever, hypertension, palpitations, peripheral vestibular syndromes

Table S4. Association between calling OHS-PC during pre-pandemic period (March-May 2019) or first COVID-19 peak and final diagnosis life-threatening disease of 3,064 callers with shortness of breath and/or chest discomfort, stratified by entrance complaint.

**Total calls**	**Pre-pandemic period (2019) n=1,224 (39.9%)**	**COVID period (2020) n=1,840 (60.1%)**	**RR (95% CI)**
LTE	162 (13.2%)	154 (8.4%)	**0.63 (0.51-0.78)**
No LTE	1,062 (86.8%)	1,686 (91.6%)	
**Shortness of breath**	**Pre-pandemic period (2019) n=631 (36.4%)**	**COVID period (2020) n=1,104 (63.6%)**	
LTE	114 (18.1%)	106 (9.6%)	**0.53 (0.42-0.68)**
No LTE	517 (81.9%)	998 (90.4%)	
**Chest discomfort**	**Pre-pandemic period (2019) n=653 (43.3%)**	**COVID period (2020) n=856 (56.7%)**	
LTE	52 (8.0%)	54 (6.3%)	0.79 (0.55-1.14)
No LTE	601 (92.0%)	802 (93.7%)	

OHS-PC: out-of-hours services for primary care.

Because some callers had both chest discomfort and shortness of breath chosen as entrance complaint, the total number of callers is lower than the number of callers with chest discomfort + the number of callers with shortness of breath
